# The experiences of giving and receiving social support for men with localised prostate cancer and their partners

**DOI:** 10.3332/ecancer.2019.989

**Published:** 2019-12-12

**Authors:** Kayleigh Nelson, Paul Bennett, Jaynie Rance

**Affiliations:** College of Human and Health Sciences, Swansea University, Singleton Park, Swansea SA2 8PP, UK

**Keywords:** prostate cancer, social support, couples, stigma

## Abstract

**Objective:**

To explore how men and their partners utilise social support in the first 12 months following a localised prostate cancer diagnosis.

**Design:**

A longitudinal qualitative design.

**Methods:**

Eighteen couples were recruited from two outpatient clinics following a localised prostate cancer diagnosis. Participants took part in semi-structured interviews at three time-points following diagnosis. Data were analysed using thematic analysis.

**Results:**

Support networks for couples became smaller as time progressed. Stigma was seen to have a role in men’s disclosure decisions. Partners generally provided higher levels of support than they received back. By Time 3, men who had previously attended social support groups rejoined to seek informational and emotional support. For partners, there appeared to be a fine line between disclosing their true feelings and protecting their partner, and they appeared to struggle to access meaningful emotional support and accept instrumental support from trusted others.

**Conclusions:**

The findings expand our understanding of the support between couples in the months following diagnosis. Social support groups were highlighted as an important source of support. Further research is now needed to help identify which couples may benefit from professional encouragement to attend these groups and which couples may benefit from alternative support provision.

## Background

Social support as a resource for people with cancer and their partners is not a new concept and may be specifically important for prostate cancer survivors who continue to report unmet emotional and informational support needs following treatment [1]. It is well-established that partners play a significant role in providing physical and emotional support throughout the illness trajectory [2–7]. Having a supportive partner can help to buffer the stress of cancer diagnosis [8, 9]. However, some studies have suggested that partners also require significant social support to enable them to provide ongoing and adequate care [3, 7].

Types of social support needs may vary over time or treatment type. In addition to the provision of tangible assistance (instrumental support or direct caregiving), other domains of social support [10, 11], such as informational (providing information to aid problem-solving), appraisal (providing information useful to self-evaluation) and emotional social support (expressions of empathy, love, caring and trust) [12], are crucial for men with prostate cancer [13]. For example, men undergoing radical prostatectomy may need more direct caregiving than men receiving radiotherapy, and men with more side effects of treatment may need more emotional support than those with fewer side effects. In addition, instrumental/caregiving support may be more essential during treatment than at the time of diagnosis; other kinds of support may be constant or vary across this diagnosis-through-treatment timeline.

To date, research has focused on the provision of support to patients by their spouses as opposed to the spouses’ own receipt of support. Supportive interaction has been associated with better recovery [13, 14], differentially associated with higher well-being in both patients and partners (e.g. [15]), and related to higher relationship satisfaction [16]. Therefore, understanding the coupled experience of giving and receiving support following a prostate cancer diagnosis may help us to identify those who are at high risk of inadequate support provision.

This study collected and analysed data from in-depth, semi-structured interviews with newly diagnosed couples to explore the kinds of social support they received, as well as reported needing or wanting but not receiving, in the first 12 months following diagnosis. Implications for long-term survivorship will be considered.

## Aim

To explore the kinds of social support men and partners received and provided in the first 12 months following a localised prostate cancer diagnosis.

## Methods

We report the findings of a prospective longitudinal qualitative study of men with newly diagnosed localised prostate cancer and their partners.

## Participants

Participants were eligible to participate in the study if: 1) they had a newly diagnosed localised prostate cancer; 2) they were in an intimate and committed relationship and 3) both members of the couple agreed to participate. Participants were required to be able to converse in English for the purpose of the interview.

## Procedure

Participants were recruited via two hospital outpatient clinics. A clinical nurse specialist screened for eligibility at the follow-up appointment (1–2 weeks after diagnosis). Those interested were introduced to the researcher (Kayleigh Nelson) who provided study information sheets and answered immediate questions. Contact details were exchanged and arrangements were made to telephone the couple in seven days, giving them time to carefully read the participant information sheet and discuss their options (see Table 1 for the topic guide).

## Data collection

Participants were interviewed at three time-points: 1) within 4 weeks of the initial diagnosis; 2) 3–4 months post-diagnosis and 3) 12 months post-diagnosis. Interviews lasted between 47 and 124 minutes and followed a semi-structured format. Most interviews took place at the participant’s home. However, for participant convenience, one Time 1 interview took place at the University and one Time 2 interview took place at the local cancer support centre. Informed consent was obtained prior to the interview.

Participants were given a choice as to whether the interviews were conducted individually or jointly as a couple at each time point. Previous research has indicated that providing couples with a choice can enhance data richness [17, 18].

## Data analysis

Interviews were recorded and transcribed verbatim using computer software Express Dictate version 5.65. Transcriptions were reviewed and coded independently by the research team.

The analysis was conducted in three parts, utilising thematic analysis approaches [19]. Individual analyses of experiences at each time point for men and partners were performed as part of a wider study. A framework to identify four domains of social support [12] was applied to the data relating to social support. Experiences of men and partners were compared and contrasted at each time point. This provided an understanding of the individual experiences, which were then brought together under an umbrella theme to represent the coupled experience.

Preliminary analyses were conducted on transcribed interviews throughout the data collection process, to help inform further interviews and ensure that important ideas were discussed and not missed. Preliminary analyses consisted of insights and observations being noted in the right-hand margin of each transcript. When interesting ideas emerged, they were added to the schedule for further interviews.

## Rigour

Two qualitative experts oversaw data analysis. Themes were critically examined to ensure dependability. To facilitate transferability, a clear description of the participants and the process of data analysis are presented.

## Ethics

This study was approved by the local NHS Research Ethics Committee (Ref no: 111217).

## Results

### Participant characteristics

Of 48 couples who met the eligibility criteria and agreed to speak to the researcher, 18 (*n* = 36) agreed to take part in this study at Time 1 (37.5% response rate). Three couples were lost to follow-up (Time 2, *n* = 2; Time 3, *n* = 1). One man (radiotherapy) withdrew from the study at Time 2 following the sudden loss of his partner. Two couples (one radiotherapy and one active surveillance) were uncontactable. Since participants consented at each time point, data from these couples were included in the analyses.

All couples were in heterosexual partnerships, though sexual preference was not a criterion for eligibility. Sample characteristics are shown in Table 2.

Eleven couples opted for individual interviews, and seven couples opted for a joint interview. No couple opted to change their preferred method for the interview, although this option was offered. In total, 82 interviews were conducted. The findings are detailed in the following sections.

## Social support across the treatment timeline

### Unmet needs

The locality of the support group appeared to be a key determinant in whether or not couples attended. Many couples who did not have access to a group locally reported wanting one.

### Instrumental support

Throughout the illness trajectory, partners provided support to men by accompanying them to hospital appointments. Men identified their partner as their primary source of support (emotional/instrumental/appraisal).

*I’ve got to say, if it wasn’t for [wife] I don’t know how I would have gotten through this, she’s been... [Becomes upset and indicates thumbs up]*. (Phillip/Radical Prostatectomy/Time 2)

## Time 1: Social support at 4 weeks post-diagnosis (prior to treatment)

### Informational/appraisal

The social support reported by couples at Time 1 was primarily a combination of informational and appraisal support. The aim was to understand prostate cancer, treatment options and side effects of the treatment to inform treatment decisions. Couples sought out social support groups because they wanted to hear directly from first-hand experiences, which helped them to make decisions about treatment:

*The support group was very good in terms of information. They weren’t experts, as they all kept pointing out, it was just helpful to know about people who have been through it and come out the other end. *(Clive/Radical Prostatectomy/Time 1)

Three couples who did not attend a support group sought information online, as well as from various healthcare professionals (e.g. specialist nurse, consultant and general practitioner), which was also perceived as being helpful.

### Emotional/information

Couples reported talking with people in their support network, mostly friends, at this point in the illness trajectory. Specifically, men sought informational support by talking with others who had experience with prostate cancer. Emotional support for men was typically provided by the partner, whereas partners turned to close friends if possible.

Many men mentioned being concerned about becoming the subject of gossip as a result of the diagnosis and their reactions varied. Most couples opting for treatment decided to disclose on a *need to know basis* (Andrew/Radiotherapy/Time 1), this included telling people whom they perceived would inevitably find out. Other men voluntarily offered this information in a bid to protect themselves from the perceived stigma associated with having cancer:

*My wife has said to me, ‘do you think you are doing right by telling people you have got cancer’, and I said, ‘why hide it?’ I think by talking about it you are getting it out in the open, like confessing a sin I suppose. I like to tell people. It gets it off your chest rather than people saying [whispers] ‘oh David’s got cancer’, ‘yeah he told me yesterday he’s got it’. You know?* (David/Radiotherapy/Time 1)

In comparison to the active treatment groups, men on active surveillance expressed a preference not to discuss their cancer once treatment decisions had been made and were pleased when their friends did not ask them about it.

## Time 2: Social support during treatment

### Instrumental support

During treatment, men having radiotherapy reported needing less instrumental support compared to men opting for a radical prostatectomy. Partners were the primary providers of instrumental support for all men and were relied upon for tasks such as lifts to/from appointments, changing dressings after surgery and cooking meals in-line with the pre-radiotherapy diet. Partners reported being happy to provide this support, and to take over household chores, in the short term, which had previously been shared by the man.

*I will have to take over more jobs which doesn’t worry me; all I want is him healthy. *(Shirley/Prostatectomy/Time 1)

### Emotional/instrumental support

Emotional support represented a common component of social support for both members of the couple during the treatment period. By this time point, most men had disclosed their diagnosis. Men receiving treatment spoke favourably about the support they had received from their partner. Some viewed disclosure outside of the relationship as a necessity, with men trying to pre-empt gossip or explain an absence from work. Some men were relieved to find that those in their wider social network were generally supportive. However, a small number of men found that people they had once perceived as friends avoided talking to them about their cancer, or in one case, avoided them altogether:

*I would say that you realise what a good family you’ve got, whereas perhaps you might have taken that for granted. And people you thought you were reasonably friendly with cross to the other side of the road to save talking to you. Well there we are, couldn’t have been a good friend in the first place*. (Andrew/Radiotherapy/Time 2)

It seemed important for partners to have an outlet: one person or a group of people who they perceived would *empathise rather than sympathise* (Sheila/Radiotherapy/Time 2) with their situation. This was primarily one or two close friends but also included adult children and work colleagues. The opportunity to talk unhindered about their situation enabled partners to work through issues and gain feelings of support and strength. However, some partners mentioned a perception that much of the support on offer was superficial, which left them unable to discuss their true experience:

*It (sexual and urinary side effects) is something that I feel is so hidden and not discussed, because you have people asking how we are and how is he, and you go ‘yeah everything is fine’ and that’s all they want to hear I think and they go ‘oh okay, that’s great, okay’ So they don’t know and they aren’t going to see the fact that, the worry of not being able to hold your wee or not having an erection. *(Steph/Radical Prostatectomy/Time 2)

In addition, some partners found it difficult to accept support from their wider network. Offers of practical help from friends went rejected and notably, partners who lived furthest from the treatment centre mentioned that, on reflection, they would have liked more emotional and instrumental support from healthcare professionals and close friends during the treatment period. Without any provisions available for them to stay nearby, it was difficult for partners of men undergoing radical prostatectomy to know what to do for the best while their spouse was in hospital; staying in a hotel or driving back and forth was costly not only financially but also emotionally and partners were left feeling tired and lonely with little support:

*I found it a lonely week because I didn’t realise there was no afternoon visiting… It’s my own fault really because I told my friend not to stay. She’s been so good, and I didn’t want to put that on her as well*. (Shirley/Radical Prostatectomy/Time 2)

*On my own I had to drive 70 miles each way... I think I stopped about 3 times before getting back here because I was so upset about leaving him. In some ways I don’t think I was fit to drive but nobody had addressed that at all. There was no welfare, for want of a better word... It was a bit shattering really and you feel left high and dry. *(Nancy/Radical Prostatectomy/Time 2)

### Social support network during treatment

By Time 2, the support network accessed by couples had become smaller and partners were the primary source of support for men. At this time point, partners generally provided higher levels of support to the man than they received back. However, partners appeared to struggle to access meaningful emotional support and accept instrumental support from trusted others. Some men reported feeling stigmatised; however, whether this was real or perceived is unclear and may be related to the man’s own historical attitudes towards cancer.

## Time 3: after treatment

### Informational/emotional support

In the months following treatment, men primarily reported seeking and receiving informational and emotional support to deal with treatment-related side effects. Men experiencing sexual dysfunction were selective about whom they spoke to, and the support group again became an important source of support. Men found it useful to speak to other men with prostate cancer experience for practical tips and emotional support. Some men mentioned a transition from needing and using support—for providing this support to other men by remaining active in prostate cancer support groups.

*As far as support afterwards, if it weren’t for the support group. They were so good. We had regular meetings and you know that every man in that room has got or has had prostate cancer. Once you’ve been to the groups, all the barriers are down, there is nothing you won’t talk about. You stand there in a pub with other men talking about erectile dysfunction which you wouldn’t have dreamed of prior. *(Robert/Radical Prostatectomy/Time 3)

*The support group was brilliant; we can go there and speak to them about anything really, and from that you get lots of useful information from others who have been through it. *(Clive/Radical Prostatectomy/Time 3)

### Emotional/appraisal

Some men mentioned building friendships with other men that they met during treatment. These friendships were helpful for both members of the couple because they provided the man with emotional support, which eased the burden on partners.

*He was in the next bed! And we are very good friends with them now. They speak every week, and they can compare notes if you know what I mean? So, that helps him. And takes a bit of the pressure off me too, really. *(Shirley/Radical Prostatectomy/Time 3)

Some partners mentioned that being the primary source of emotional support was becoming increasingly tiring. They also expressed difficulties in obtaining emotional support for themselves. Many partners reported being unable to talk openly and honestly with their closest friends due to a perceived lack of understanding from others about ongoing prostate-cancer related issues. At the same time, partners were cautious of disclosing too much information in a bid to protect the men with prostate cancer:

I think it’s hard because to all intents and purposes to everybody else, he’s better. I think there are some people who are aware of those two issues still ongoing, but they are not aware of how I truly feel because I haven’t expressed that... for him really. (Steph/Radical Prostatectomy/Time 3)

In contrast, couples on active surveillance reported having very little to discuss in terms of their prostate cancer and those who had told their close friends perceived them to be supportive but were pleased that their friends did not constantly talk about it:

*Some of our closest friends know. They have the same ideas as us really. Nobody says a lot about any of their ailments. Everybody knows but uh, actually, we don’t sit down and talk about our illnesses. For me, there is nothing to say. I think I would be concerned if people kept bringing it to my attention. *(Hugh/Active Surveillance/Time 3)

### Unmet need

While most men receiving radical treatment said that their friends were helpful, and partners were sympathetic, the need for access to one-to-one emotional support from professionals was a strong theme running through the data. This was primarily, but not solely, reported by men who had limited access to peer support:

*Follow-up phone calls would be nice. By people who know what they are talking about. Pre-empting problems rather than reacting. Just pre-empt the problems you are going to get and why. You get all of these weird emotions... more contact would be nice*. (Charles/Radiotherapy/Time 3)

### Social support network at Time 3

By this time point, men still perceived their partner as their primary source of support. Men who had previously attended social support groups rejoined to seek informational and emotional support. For partners, there appeared to be a fine line between disclosing their true feelings and protecting their partner which made it more difficult for them to obtain meaningful social support.

## Discussion

In this longitudinal qualitative study of 18 couples facing prostate cancer, there are two key findings. First, for the men in this sample, partners were their primary source of support throughout the illness trajectory. Men also obtained informational and emotional support from prostate cancer support groups at diagnosis and after treatment, which they perceived as being helpful. Some partners and men, however, expressed difficulties in obtaining social support. For men, this was primarily post-treatment, whereas for partners, difficulties initiating support and sustaining support increased over time, often due to the physical demands of providing care to their ill partner and concern about disclosing intimate details to mutual friends. Some men preferred to discuss their concerns with healthcare professionals rather than friends or family. Second, stigma has a role in men’s disclosure decisions. Of these men, some chose to disclose widely in an attempt to protect themselves from gossip, while others were more selective and disclosed on a need to know basis. Men perceived that friends treated them differently once they had disclosed their diagnosis. This has the potential to increase isolation for the men concerned, possibly depriving them of a supportive network.

## Comparison to prior literature

This study clearly articulates how couples’ social networks influence the availability and receipt of social support. Overall, the men in this sample were satisfied by the support they received from their partner but would have liked more emotional support from healthcare professionals as time went on. Those who attended support groups found them invaluable sources of information and having someone else for the man to speak to was helpful for some partners. The experiences of men in this study fit well within the dominant paradigms of social support domains [12]. For example, the type of social support sought, following the diagnosis and before treatment, was largely informational and true to the original definitions of informational support; it was sought for the purpose of treatment decision making.

The stigma felt by many of the men in this sample appeared to be largely due to personal attitudes towards cancer. Perceived stigma was found to influence some participant’s disclosure regarding prostate cancer and also appeared to prevent others from raising related issues with men and partners; both of which are likely to hinder support seeking [20]. This is partly consistent with findings from two previous qualitative studies which suggest that men with prostate cancer are reluctant to disclose due to stigma associated with treatment side effects [21, 22]. On the other hand, some men engaged in indiscriminate disclosure in an attempt to protect themselves from the stigma.

Consistent with the view that perceived stigma may act as a barrier to help-seeking (based on findings in the general population) [23, 24], there were indications in this study that stigma has the potential to impair the help-seeking behaviour of men with prostate cancer and their partners.

Our findings appear consistent with the view that the woman’s role as emotional caregiver in the male–female dyad has emotional costs. Previous research has reported that female partners are reluctant to share their distress with their husband in order to minimise the stress of the illness and to avoid discussing issues that create emotional tension [25]. The findings of this study show that partners find it difficult to discuss concerns with close friends. This means that partners often have to deal with their distress and anxiety alone, and is consistent with other studies [26]. Consistent with other literature, the men in this study relied on their partner for emotional support, and this means that partners have to manage not only their own anxiety but also the distress of their husbands [25]. Furthermore, our findings suggest that support between men and partners becomes inequitable during treatment and remains the same 6 months later (in contrast to [16]). While perceptions of stigma were not cited as direct causes of social isolation, partner’s reluctance to disclose prostate-related issues to others likely contributed to the degree of social isolation they reported feeling.

## What does this mean for long-term survivorship?

There is ample evidence that a lack of social support during times of need can be stressful, particularly for those with insufficient opportunities to obtain it. Although the exact mechanism linking social support with health-related outcomes remains unclear, social support seems to play an important role in living with cancer [27, 28].

Untapped support resources may exist in couples’ informal social networks. While most couples did not want to talk incessantly about prostate cancer, having confidence in the availability of adequate, meaningful support is important [1]. Our findings indicate that couples may require more support towards the end of the treatment trajectory (around 12 months post-diagnosis). Many couples commented on the usefulness of attending social support groups to connect and share experiences with peers during this time (in line with [29]). However, social support groups do not appeal to everyone. Further research is needed to help identify which couples may benefit from professional encouragement to attend these groups and which couples may benefit from alternative support provision.

In addition, our study highlights that perceived cancer-related stigma has the potential to impair the couple’s ability to access high-quality social support. Research to further explore this link may reveal opportunities for supportive interventions in these couples.

## Study limitations

While recruitment was relatively high, the ethics of recruiting participants close to diagnosis meant that the sample was self-selected; therefore, couples using more avoidant behaviours may not have elected to take part in the study. Also, while aiming to be inclusive of race and sexual orientation, the couples in this study were predominantly white-British, heterosexual and in long-term relationships. The findings must be interpreted with this in mind.

## Conclusions

The findings of this study expand our understanding of the support between couples in the months following diagnosis. Social support groups were highlighted as an important source of support for men. Further research is now needed to help identify which couples may benefit from professional encouragement to attend these groups and which couples may benefit from alternative support provision.

## List of abbreviations

None.

## Conflicts of interest

The author(s) declare that they have no conflicts of interest.

## Disclosure of results at a meeting

The findings reported in this paper were previously presented at the British Psychological Society’s Division of Health Psychology Annual Conference in 2017.

## Figures and Tables

**Table 1.** Interview topic guide.

Participants were asked:
To describe the experience of being (or their partner being) diagnosed with localised prostate cancer.
How they felt about localised prostate cancer.
Whether there had been any aspect of the diagnosis or treatment decision-making that had been particularly difficult.
To consider whether there had been any positive aspects of the experience.
How they coped with localised prostate cancer on a daily basis and what concerns they had, if any.

**Table 2.** Demographics and characteristics of the sample (n = 36).

		*n* (%) Time 1	Time 2	Time 3
**Men**				
Age at diagnosis (years)	50–59 60–69 70–79	2 (11.1) 9 (50) 7 (38.8)	2 (12.5) 8 (50) 6 (37.5)	2(13.3) 7 (46.6) 6 (40)
Employment	Self-employed Unskilled Retired	4 (22.2) 1 (5.6) 13 (72.2)	3 (18.75) 0 (0) 13 (86.6)	3 (20) 0 (0) 12 (80)
Route to prostate specific antigen test	Lower urinary tract symptoms Other health problem Annual health check	10 (55.5) 5 (27.7) 3 (16.6)	8 (50) 5 (31.2) 3 (18.7)	8 (53.3) 4 (26.6) 3 (20)
Type of treatment chosen	Active surveillance Radical prostatectomy Hormone/radiotherapy	5 (27.7) 6 (33.3) 7 (38.8)	4 (25) 6 (37.5) 6 (37.5)	3 (20) 6 (40) 6 (40)
**Partners**				
Age (years)	50–59 60–69 70–79	8 (44.4) 8 (44.4) 2 (11.1)	8 (50) 7 (43.8) 1 (6.3)	7 (46.6) 7 (46.6) 1 (6.7)
Employment	Professional Unskilled Retired	2 (11.1) 4 (22.2) 12 (66.7)	2 (12.5) 3 (18.8) 11 (68.8)	2 (13.3) 3 (20) 10 (66.7)
**Couples**				
Relationship length	<15 years 15–24 years 25–34 years 35+ years	2 (11.1) 5 (27.7) 7 (38.8) 4 (16.6)	2 (12.5) 4 (25) 7 (43.7) 3 (18.75)	2 (13.3) 4 (26.6) 6 (40) 3 (20)
Past experience of coping with serious illness as a couple	Yes No	7 (38.8) 11 (61.1)	7 (43.7) 9 (56.2)	6 (40) 9 (60)

Three couples were lost to follow-up (T2, *n* = 2 [radiotherapy and active surveillance]; T3, *n* = 1[radiotherapy]). None of the men on active surveillance commenced active treatment during the study period.

## References

[1] King AJ, Evans M, Moore T (2015). Prostate cancer and supportive care: a systematic review and qualitative synthesis of men’s experiences and unmet needs. Eur J Cancer Care (Engl).

[2] Matsunaga D, Gotay C (2004). Characteristics contributing to an enduring prostate cancer support group in an Asian and Pacific islander community. J Psychosoc Oncol.

[3] Boehmer U, Babayan R (2005). A pilot study to determine support during the pre‐treatment phase of early prostate cancer. Psycho-Oncology.

[4] Ervik B, Nordoy T, Asplund K (2010). Hit by waves – living with local advanced or localised prostate cancer treated with endocrine therapy or under active surveillance. Cancer Nurs.

[5] Walsh E, Hegarty J (2010). Men’s experiences of radical prostatectomy as treatment for prostate cancer. Eur J Oncol Nurs.

[6] O’Shaughnessy P, Laws T, Esterman A (2013). The prostate cancer journey. Results of an online survey of men and their partners. Cancer Nurs.

[7] Rivers B, August E, Quin G (2012). Understanding the psychosocial issues of African American couples surviving prostate cancer. J Cancer Educ.

[8] Kayser K, Scott J (2008). A dyadic approach to coping with women’s cancers. Helping Couples Cope with Women’s Cancers: An Evidence-Based Approach for Practitioners.

[9] Manne S, Ostroff J, Winkel G (2005). Partner unsupportive responses, avoidant coping, and distress among women with early stage breast cancer: patient and partner perspectives. Health Psychol.

[10] Berkman L (2000). Social support, social networks, social cohesion and health. Soc Work Health Care.

[11] Cohen S (2004). Social relationships and health. Am Psychol.

[12] Berkman L, Glass T (2000). Social integration, social networks, social support and health. Social Epidemiology.

[13] Voerman B, Visser A, Fischer M (2007). Determinants of participation in social support groups for prostate cancer patients. Psycho-Oncology.

[14] Pinquart M, Duberstein P (2010). Associations of social networks with cancer mortality: a meta-analysis. Crit Rev Oncol.

[15] Kuijer R, Buunk B, Ybema J (2002). The relation between perceived inequity, marital satisfaction and emotions among couples facing cancer. Br J Soc Psychol.

[16] Knoll N, Scholz U, Burkert S (2009). Effects of received and mobilized support on recipients’ and providers’ self-efficacy beliefs: a 1-year follow-up study with patients receiving radical prostatectomy and their spouses. Int J Psychophysiol.

[17] Morris S (2001). Joint and individual interviewing in the context of cancer. Qual Health Res.

[18] Taylor B, de Vocht H (2011). Interviewing separately or as couples? Considerations of authenticity of method. Qual Health Res.

[19] Braun V, Clarke V (2006). Using thematic analysis in psychology. Qual Res Psychol.

[20] Major B, O’Brien LT (2005). The social psychology of stigma. Annu Rev Psychol.

[21] Gray RE, Fitch M, Phillips C (2000). To tell or not to tell: patterns of disclosure among men with prostate cancer. Psycho-Oncology.

[22] Ettridge K, Bowden J, Chambers S (2018). ‘Prostate cancer is far more hidden...’: perceptions of stigma, social isolation and help-seeking among men with prostate cancer. Eur J Cancer Care (Engl).

[23] Braybrook D, Witty K, Robertson S (2011). Men and lung cancer: a review of the barriers and facilitators to male engagement in symptom reporting and screening. J Mens Health.

[24] Chapple A, Ziebland S, McPherson A (2004). Stigma, shame, and blame experienced by patients with lung cancer: qualitative study. BMJ.

[25] Boehmer U, Clark J (2001). Communication about prostate cancer between men and their wives. J Fam Pract.

[26] Couper J, Bloch S, Love A (2006). Psychosocial adjustment of female partners of men with prostate cancer: a review of the literature. Psycho-Oncology.

[27] Cobb S (1976). Social support as a moderator of life stress. Psychosom Med.

[28] Cohen S (1988). Psychosocial models of the role of social support in the etiology of physical disease. Health Psychol.

[29] Oliffe J, Chambers S, Garrett B (2015). Prostate cancer support groups Canada-based specialists perspectives. Am J Mens Health.

